# Informing Endpoints for Clinical Trials of Geographic Atrophy

**DOI:** 10.1146/annurev-vision-101922-045110

**Published:** 2024-09

**Authors:** Eleonora M. Lad, Monika Fleckenstein, Frank G. Holz, Liangbo Shen, Lucian V. Del Priore, Rufino Silva, Giovanni Staurenghi, Nadia Waheed, Usha Chakravarthy

**Affiliations:** 1 Department of Ophthalmology, Duke University Medical Center, Durham, North Carolina, USA; 2 Department of Ophthalmology and Visual Science, John A. Moran Eye Center, University of Utah, Salt Lake City, Utah, USA; 3 Department of Ophthalmology, University of Bonn, Bonn, Germany; 4 Department of Ophthalmology, University of California, San Francisco, California, USA;; 5 Department of Ophthalmology and Visual Science, Yale School of Medicine, New Haven, Connecticut, USA; 6 Faculty of Medicine, University of Coimbra (FMUC), Coimbra, Portugal; 7 Department of Ophthalmology, Coimbra Hospital and University Centre (CHUC), Coimbra, Portugal; 8 Clinical Academic Centre of Coimbra (CACC), Coimbra, Portugal; 9 Association for Innovation and Biomedical Research on Light and Image (AIBILI), Coimbra, Portugal; 10 Department of Biomedical and Clinical Sciences, Luigi Sacco Hospital, University of Milan, Milan, Italy; 11 Tufts Medical Center, Boston, Massachusetts, USA; 12 Department of Ophthalmology, Center for Public Health, Queen’s University, Belfast, Northern Ireland, United Kingdom

**Keywords:** geographic atrophy, structural endpoints, functional endpoints, microperimetry, low-luminance visual acuity, reading speed, age-related macular degeneration

## Abstract

Geographic atrophy (GA), the non-neovascular advanced form of age-related macular degeneration, remains an important disease area in which treatment needs are currently unmet. Recent clinical trials using drugs that target the complement pathway have shown modest yet consistent reductions in GA expansion but without commensurate changes in measures of visual function. In this review, we summarize information from the wide range of studies describing the characteristics of GA morphology and enumerate the factors influencing the growth rates of lesions and the directionality of expansion. In addition, we review the relationship between GA growth and the various measures of vision that reflect changes in function. We consider the reasons for the discordance between the anatomical and functional endpoints in current use and discuss methods to align these key outcomes.

## 1. INTRODUCTION

### 1.1. Natural History of Geographic Atrophy and Outcomes

Age-related macular degeneration (AMD) is the third most prevalent cause of severe and irreversible vision loss globally (Fleckenstein et al. 2018, Sunness et al. 2007). Projections indicate that, by the year 2040, the count of individuals impacted by AMD will hover around 300 million, a statistic that carries substantial socioeconomic implications (Holz et al. 2007). AMD is a multifactorial disease caused by a combination of genetic and environmental factors and in which oxidative stress, disruption of the complement system, and inflammation are believed to play key pathophysiologic roles in disease development and progression (Domalpally et al. 2013, Feuer et al. 2013, Sunness et al. 1999). The initial stages of AMD are marked by the accumulation of drusen, along with modifications in the retinal pigment epithelium (RPE) and pigment accumulation. Progression to the advanced stages can take the form of neovascular (also termed as wet or exudative) or non-neovascular (referred to as atrophic, dry, or nonexudative) presentations (Shen et al. 2021b). The atrophic manifestation of advanced AMD is known as geographic atrophy (GA) and is characterized by the irreversible loss of retinal photoreceptors, RPE, and choriocapillaris (Schmitz-Valckenberg et al. 2016).

The clinical presentation of GA is variable, with unifocal or multifocal and foveal or extrafoveal lesions that typically enlarge over time (Lindner et al. 2015b, You et al. 2021). The fundamental mechanisms driving GA lesion progression and its inherent natural history are not fully understood. Within clinical investigations, the average rates of lesion advancement exhibit significant diversity across individuals, spanning from 1.2 to 2.8 mm^2^ per year (Feuer et al. 2013, Shen et al. 2020), with numerous risk factors modulating GA expansion. These include subretinal drusenoid deposits, hyporeflective cores in drusen, intraretinal hyperreflective foci, choriocapillaris flow deficits, specific patterns of hyperautofluorescence, and the type of macular neovascularization if it is concomitantly present in the same or contralateral eye (Allingham et al.2016, Biarnés et al. 2015, Fleckenstein et al. 2011b, Giocanti-Auregan et al. 2015, Moussa et al. 2013, Schmitz-Valckenberg et al. 2006, You et al. 2021, Zhang et al. 2022).

In the early stages of AMD, patients commonly experience alterations in visual function characterized by a delay in dark adaptation; diminished sensitivity to contrasts; and, subsequently, the appearance of scotomas in their central visual field. As the area of atrophy increases to involve larger areas of the central retina, patients begin to experience a more pronounced deterioration in central visual acuity, slower reading speed, distorted vision, challenges in discerning colors, extremely slow recovery in dark adaptation following exposure to bright light, and substantial impairment of overall visual function (Feuer et al. 2013). This gradual decline of visual capabilities underscores the insidious nature of GA, where irreversible visual loss occurs incrementally and profoundly impacts individuals’ ability to perform daily activities and maintain the same quality of life. In particular, the median time to the loss of 15 letters [three Early Treatment Diabetic Retinopathy Study (ETDRS) lines] is 3 years (Pfau et al. 2020b). In a study of the natural history of GA, the cumulative risk of a six-line decrease in best-corrected visual acuity (BCVA) at 2 years was 21%, and the cumulative risk of legal blindness at 4 years was 27%, in patients who presented with BCVA better than 20/50 (Stetson et al. 2014).

The current primary outcome measurements in clinical trials for GA are assessments of the area of the atrophic regions along with measures of vision including BCVA, reading speed, and low-luminance visual acuity (LLVA), as well as patient-reported outcome measures. BCVA evaluates the performance of the fovea in discerning individual letters of a given size, whereas fluent reading requires a larger area of the central macula to perform well. Reduced reading speed impacts activities of daily living that require reading (Laiginhas et al. 2022, Lee et al. 2013). Visual function questionnaires offer quantitative assessments of an individual’s visual capabilities, influenced not only by the condition of their eyes, but also by their physical, emotional, and cognitive well-being. One such instrument, the National Eye Institute Visual Function Questionnaire-25 (NEI-VFQ-25), is widely utilized and has been found to have good reliability and construct validity, particularly in patients with neovascular AMD (Künzel et al. 2020).

Because GA expands over time, a robust assessment of the change in area and location with regard to the fovea is essential. For this reason, several imaging modalities are used to define the margin of the GA lesions. Color fundus photography has been the historical gold standard for assessment of GA area, but although it is still used, it has largely been supplanted by fundus autofluorescence (FAF) as a marker of outcome. FAF offers superior contrast and clarity for assessing the borders of atrophic regions (Shen et al. 2020, Thorell et al. 2015). In blue-light FAF, regions of atrophy appear as well-demarcated areas of decreased signal intensity, typically surrounded by an area of increased FAF signal (Nattagh et al. 2020). However, the central macular luteal pigment absorbs the blue excitation light, resulting in a hypoautofluorescent signal in normal eyes, making assessment of foveal involvement by GA challenging. For this reason, green FAF is used, which eliminates the confounding effect of absorption by macular pigment. Recently, en face analysis of optical coherence tomography (OCT) has been proposed as an alternative method; using this method, regions of photoreceptor and RPE loss are seen as hypertransmissions of signal [hypertransmission defects (HTDs)] into the choroid (Moult et al. 2021, Nassisi et al. 2019, Sacconi et al. 2021). En face assessment represents a potential tool to identify minute foci of persistent choroidal HTDs that progress into much larger lesions that are detectable by color and/or autofluorescence imaging and recognizable as typical GA. Thus, longitudinal analysis of such persistent HTDs could be used as an endpoint in clinical trials investigating new therapies for atrophic AMD (Capuano et al. 2017, Pfau et al. 2020a).

### 1.2. Treatment Landscape

The largest Phase 3 trial to date to evaluate the complement factor D inhibitor lampalizumab reported no differences in GA enlargement versus sham during 48 weeks of treatment. Nonetheless, the findings from Chroma and Spectri served to highlight the substantial and consistent enlargement of GA, at a mean of approximately 2 mm^2^ per year (Heiferman & Fawzi 2019). In a Phase 2 study (ClinicalTrials.gov identifier NCT00658619), the brimonidine drug delivery system (DDS) treatment was well tolerated and appeared to slow GA lesion growth, particularly in patients with lesions 6 mm^2^ or larger at baseline (Carnevali et al. 2016). However, the planned Phase 3 programs involving brimonidine DDS have been discontinued, rendering any further assessment and conclusions unattainable.

Two drugs that target the complement pathway recently gained approval by the US Food and Drug Administration (FDA) for treatment of GA. The Phase 2 FILLY study, which evaluated pegcetacoplan, a complement component C3 inhibitor, showed a reduction in the rate of progression of GA at month 12 (Roisman et al. 2016). Subsequently, two pivotal Phase 3 trials, OAKS (ClinicalTrials.gov identifier NCT03525613) and DERBY (ClinicalTrials.gov identifier NCT03525600), confirmed a reduction in GA lesion growth in pegcetacoplan-treated eyes compared to sham over the 24-month period. Despite an average reduction of GA growth by approximately 20%, no similar benefit was observed in any of the prespecified visual function endpoints. Pegcetacoplan was approved by the FDA in February 2023. The GATHER trials tested avacincaptad pegol, which specifically inhibits complement C5 while preserving the activity of complement C3, thereby offering potential safety advantages in the context of complement-related disorders (Querques et al. 2013). These trials found significant reductions in GA growth rate over 12 months and a marginal favorable trend (though not significant) for BCVA, with no benefit noted for LLVA. Avacincaptad pegol was approved by the FDA in August 2023. The finding that two modifiers of the complement pathway in two separate trial programs resulted in modest effects on inhibition of GA growth are promising, although functional outcomes indicate no benefit over placebo.

The purpose of this review is to consider the factors underpinning the lack of agreement betweenthemorphology(structure)andfunctionofGAtoaidinfutureclinicaltrials.Understanding these factors is essential, as it can provide valuable insights into the nature of the condition and potentially guide improvements in diagnosis, treatment, or patient care.

## 2. GEOGRAPHIC ATROPHY PROGRESSION AND MODIFYING FACTORS

**Figure 1:**
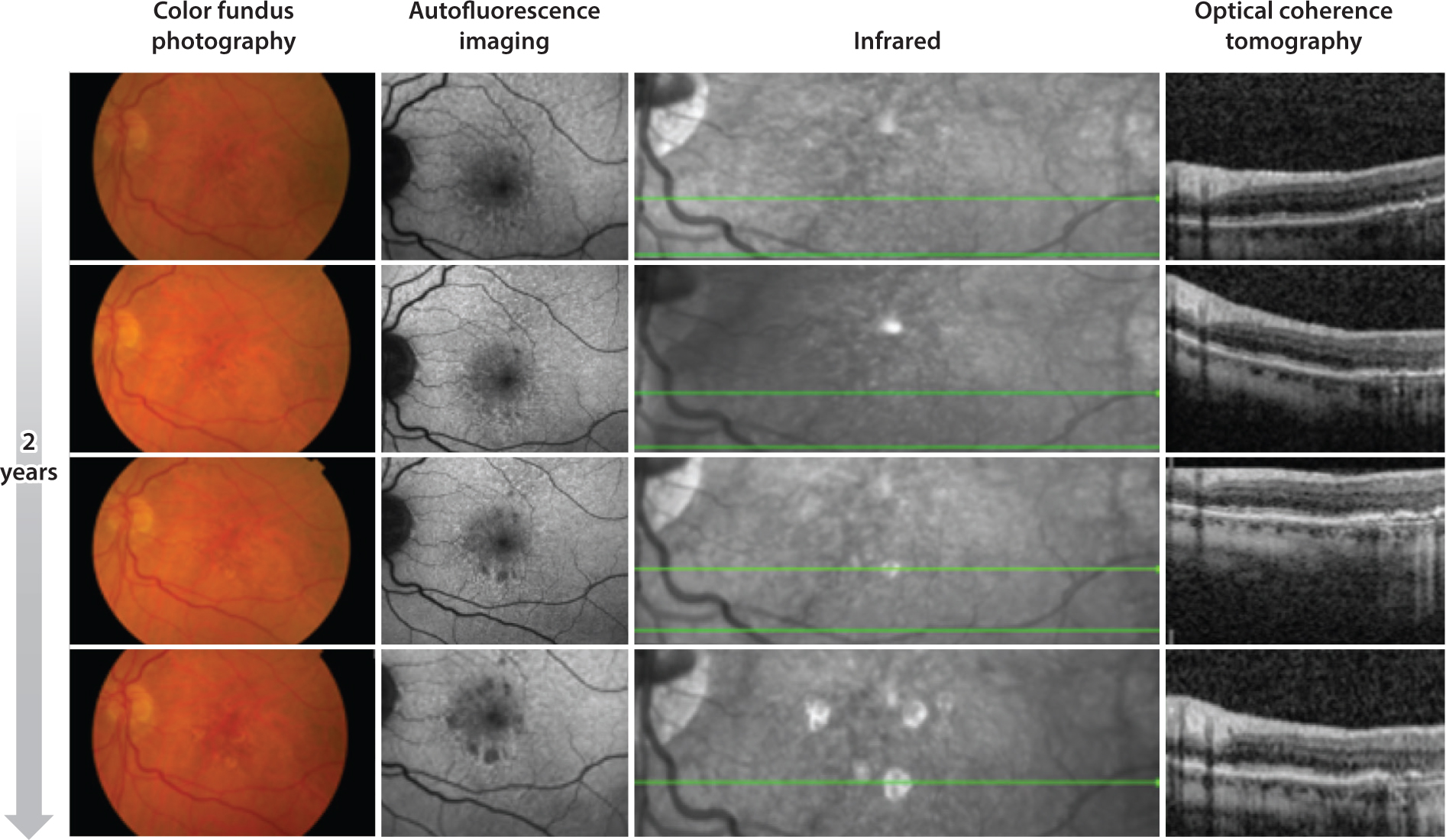
Multimodal longitudinal imaging of an eye with intermediate age-related macular degeneration, retinal pseudodrusen, and incomplete retinal pigment epithelial and outer retinal atrophy (iRORA) that progressed to geographic atrophy over a 2-year interval. From left to right: color fundus photography, autofluorescence imaging, infrared, and optical coherence tomography.

The goal of this section is to describe the predictors of GA progression, including specific lesion characteristics on imaging [FAF, OCT, OCT angiography (OCT-A)] (Figure 1); fellow eye features; genetic, environmental, and demographic factors; and the combination of prognostic factors.

### 2.1. Lesion Features and Specific Characteristics of the Affected Eye

The size of the GA lesion at baseline consistently correlates with its progression rate, with smaller baseline lesions being associated with lower rates of progression (Fleckenstein et al. 2018). In the natural history study by Sunness and coworkers (2007), lesions of less than 1.3 mm^2^, 1.3–8.3 mm^2^, and 8.3 mm^2^ or more exhibited progression rates of 0.8 mm^2^/year, 2.1 mm^2^/year, and 3.0 mm^2^/year, respectively. Similarly, the Fundus Autofluorescence in Age-Related Macular Degeneration (FAM) study demonstrated that the median progression rate of the lowest baseline size quartile [0.74 mm^2^/year for lesions less than 1 disc area (DA), which is equivalent to 2.54 mm^2^] was significantly lower than that of larger lesion quartiles (1–3 DA: 1.56 mm^2^/year; 3–5 DA: 1.80 mm^2^/year; 5–10 DA: 1.88 mm^2^/year) (Holz et al. 2007). However, it remains unclear whether the association between baseline lesion size and progression rate is a prognostic indicator for an individual’s disease progression, or if it reflects the heterogeneity of disease within a study cohort, where baseline lesion sizes can range considerably.

Some studies used mathematical strategies such as the square-root transformation (SQRT) of baseline lesion size to account for the skewness of data (Feuer et al.2013). When the SQRT transformation was applied to the Age-Related Eye Disease Study (AREDS) data set, the association between baseline lesion size and progression rate was no longer statistically significant (Feuer et al. 2013).

Eyes with multifocal lesions have GA enlargement rates significantly higher than those of eyes with unifocal lesions (Fleckenstein et al. 2018). Sunness et al. (1999) reported that eyes that transitioned from unifocal lesions to multifocal, horseshoe, ring, or solid configurations demonstrated greater rates of GA growth than eyes with a stable lesion configuration (Sunness et al. 1999). To objectively measure this observation, researchers introduced the GA circularity index (GACI), which takes into account the perimeter and deviation from circularity of GA lesions (Domalpally et al. 2013). Eyes with lower GACI values, indicating lesions that deviate more from a circular shape, generally consisted of multifocal lesions with higher rates of expansion compared to eyes with higher GACI values (Domalpally et al. 2013).

In a recent meta-analysis, Shen et al.(2021b) concluded that the effective radius of GA enlarges linearly and steadily over time in both unifocal and multifocal GA. Additionally, lesion focality was identified as a significant prognostic factor for the progression rate of the GA effective radius. They proposed that expansion of GA area is directly proportional to the total lesion perimeter, which serves as a measure of the number of retinal pigment epithelium cells exposed at the lesion border (Shen et al. 2021b).

The enlargement of GA lesions differs depending on their location. In the Geographic Atrophy Progression study, it was observed that extrafoveal lesions enlarge at significantly greater rates compared to foveal lesions (Schmitz-Valckenberg et al. 2016). In an analysis of the directional progression kinetics among patients in the FAM study who had baseline foveal sparing, it was found that lesion progression toward the periphery was 2.8-fold faster than progression toward the fovea. The rates were calculated as 0.319 mm/year (SQRT) for peripheral progression and 0.116 mm/year (SQRT) for foveal progression (Lindner et al.2015b). These findings were further supported by You et al. (2021), who confirmed that the pace of progression increases as the distance from the foveal center increases. However, in a systematic review and meta-analysis, Shen et al. (2020) showed that GA expansion was greater in extrafoveal lesions compared to subfoveal lesions. They also observed that, within the macula (0–3,000 μm from the foveal center), the GA effective radius increased continuously with increasing retinal eccentricity. Interestingly, beyond the macular region (>3,600 μm from the foveal center), they reported a dramatically decreased progression (Shen et al. 2020), although this finding may be confounded by their inability to accurately measure large GA lesions that extend the image frame or coalesce with peripapillary atrophy.

### 2.2. Fundus Autofluorescence Imaging Characteristics

In most eyes, the dark, hypoautofluorescent patches indicating GA lesions are surrounded by varying degrees of hyperautofluorescent alterations, particularly at junctional regions of atrophy. Notably, studies have shown a positive correlation between GA progression rates and the extent of hyperautofluorescence surrounding the atrophic lesion (Allingham et al. 2016, Schmitz-Valckenberg et al. 2006).

The FAM study investigated the correlation between perilesional FAF hyperautofluorescence patterns and GA progression rates (Holz et al. 2007). FAF patterns were classified as none, focal, banded, patchy, or diffuse; diffuse patterns were further categorized as reticular, branching, fine-granular, fine-granular with peripheral punctate spots, or trickling. The GA progression rate was associated with FAF patterns, with the least expansion observed in eyes with none or focal patterns, and the most expansion observed in eyes with banded or diffuse patterns. Eyes with the diffuse-trickling pattern represented a subgroup with particularly rapid progression (Holz et al. 2007). This relationship between FAF patterns and GA progression rate has been replicated in other cohorts (Fleckenstein et al. 2018).

However, FAF patterns are associated not only with the GA progression rate, but also with baseline lesion size and follow-up duration (Biarnés et al. 2015). In one study, eyes with a focal pattern or no perilesional FAF changes had smaller baseline GA areas than eyes where the perilesional areas exhibited banded or diffuse FAF patterns. The authors of this study concluded that FAF patterns reflect both disease severity and duration and may evolve from no perilesional or focal FAF to diffuse or banded patterns (Biarnés et al. 2015).

### 2.3. Optical Coherence Tomography Features

OCT scans of GA lesions can show structural abnormalities at the junctional zone, including irregular RPE elevations and splitting of the RPE–Bruch’s membrane (BM) complex. These features are associated with faster progression rates of GA compared with lesions with smooth margins (Moussa et al. 2013). Splitting of the RPE–BM complex is also seen in eyes with the rapid-progressing diffuse-trickling FAF phenotype (Fleckenstein et al. 2011b).

Outer retinal layer thickness showed a significant negative correlation with annual GA progression but also showed a significant correlation with the RPE–BM distances, suggesting that these distances were dependently associated with GA progression (Zhang et al. 2022).

A study assessing the ellipsoid zone (EZ) using en face OCT found that 43% of eyes demonstrated a pattern of disruption outside of the baseline GA lesion that predicted the 1-year location of GA progression (Giocanti-Auregan et al. 2015). A recent analysis of the Directional Spread of GA (DSGA) study found that the distance between EZ loss and the GA boundary and photoreceptor outer segment thickness were prognostic for future progression rates. Outer nuclear layer and photoreceptor inner segment thinning over time was significant even when adjusting for age and proximity to the GA boundary (Pfau et al. 2020b).

Minimum intensity (MI) is the lowest image intensity from each A-scan from the sub-RPE slab region. Excluding the fovea, MI values were significantly higher in areas of lesion progression and correlated with overall progression rate. Areas of increased MI corresponded to hyperreflectivity, or atrophy, of the outer nuclear or Henle fiber layers on cross-sectional scans (Stetson et al. 2014).

HTDs detected on en face OCT images were shown to serve as an early standalone OCT biomarker for the future formation of GA (Laiginhas et al. 2022). In their study in patients with intermediate AMD, Laiginhas et al. (2022) estimated an 80-fold-increased risk of developing GA once an HTD ≥250 μm appeared (Laiginhas et al. 2022).

Reduced subfoveal choroidal thickness has been correlated with higher GA progression rates (Lee et al. 2013). In addition, the fast-progressing diffuse-trickling FAF pattern has been found to have a significantly thinner choroid than non-diffuse-trickling phenotypes. Overall, eyes with GA have reduced subfoveal choroidal thickness compared with age-matched healthy eyes (Adhi et al. 2014, Lindner et al. 2015a, Thorell et al. 2015), although one study reported that this reduction was limited to the group of eyes with reticular pseudodrusen (Thorell et al. 2015).

### 2.4. Optical Coherence Tomography Angiography Features

Several studies have described increased choriocapillaris rarefication up to total loss with the presence of larger choroidal vessels directly adjacent to the BM in areas of GA. OCT-A may reveal attenuated choriocapillaris flow signal in patients with GA, even in areas not affected by GA. Moreover, the area surrounding the GA margin may show a significantly higher flow impairment compared with more distant areas in the same eye, and this flow impairment seems to correlate with the year-to-year progression rate of GA (Nassisi et al. 2019, Nattagh et al. 2020, Sacconi et al. 2021). However, another study did not provide support for a strong correlation between local, OCT-A-measured choriocapillaris flow deficits and local GA progression rates (Moult et al. 2021). Nevertheless, the authors of the latter study emphasized that this does not preclude the existence of a correlation between local physiological choriocapillaris flow impairments and local GA progression rates (Moult et al. 2021).

Several studies have demonstrated reduced GA progression in the presence of or proximity to type 1 macular neovascularization (MNV) lesions (Capuano et al. 2017, Christenbury et al. 2018, Dansingani & Freund 2015, Heiferman & Fawzi 2019, Pfau et al. 2020a). Type 1 MNV is associated with a localized, dense vascular network between the BM and the RPE on OCT-A (Carnevali et al. 2016, Querques et al. 2013, Roisman et al. 2016) and a double-layer or shallow irregular RPE elevation (SIRE) sign on structural OCT (Narita et al. 2020, Sato et al. 2015, Shi et al. 2019). Similarly, a recent subanalysis of AREDS2 demonstrated that GA enlargement before the development of exudative MNV was slowed, indicating that perilesional nonexudative MNV tissue may have slowed the enlargement of smaller GA lesions through improved perfusion (Hwang et al. 2021).

These studies support the model that MNV development is intrinsically a protective mechanism (Grossniklaus & Green 2004, McLeod et al. 2009, Sarks et al. 1997). Indeed, type 1 MNV could be regarded as a vascular patch in the sub-RPE space that has overcome AMD-related changes in the BM to take over the task of the choriocapillaris in direct proximity to the RPE and photoreceptors (Fleckenstein et al. 2021).

### 2.5. Other Characteristics of the Affected Eye

Vitreoretinal traction on OCT has been reported to be associated with GA progression. Mechanical stress of vitreoretinal traction may affect the natural history of GA, and it has been hypothesized that this stress could lead to structural distortion of the RPE layer (Abdillahi et al. 2014).

A recent analysis in the AREDS2 cohort confirmed that GA progression is faster in eyes with reticular pseudodrusen (Agrón et al. 2023). Interestingly, in this analysis, the presence of reticular pseudodrusen and the *ARMS2/HTRA1* genotype were relatively independent risk factors, operating via distinct mechanisms.

### 2.6. Fellow Eye Characteristics

Generally, there is a high correlation in the progression rates between the two eyes among patients with bilateral GA, although individual variability exists (Fleckenstein et al. 2018). The status of the fellow eye’s disease also plays a role in the progression. When the fellow eye has GA (bilateral GA), GA tends to progress at higher rates. Conversely, when the fellow eye has early or intermediate AMD, the progression rates are lower. Intermediate rates of progression are observed when the fellow eye has choroidal neovascularization (Fleckenstein et al. 2011a, Grassmann et al. 2015, Sunness et al. 2007).

### 2.7. Genetic, Environmental, and Demographic Factors

While numerous studies have identified genetic, environmental, and demographic factors associated with the development of GA, these factors have differential impacts on GA progression. Keenan (2023) recently conducted a comparative analysis of risk and protective factors for development of GA versus GA lesion progression (expansion). The findings revealed that certain factors are shared, meaning that they operate in the same direction at both stages. Other factors are not shared, while some seem to have different effects at each stage: Risk variants at *ARMS2/HTRA1* increase both the risk of progression to GA and the rate of GA expansion, presumably through the same mechanism. On the other hand, risk and protective variants at *CFH/CFHR* alter the risk of developing GA but do not influence the rate of GA expansion. A risk variant at *C3* increases the risk of GA but is associated with slower GA expansion. Among environmental factors, cigarette smoking is associated with an increased risk of GA and faster GA expansion, whereas age is associated with increased risk but not with the rate of GA expansion. The Mediterranean diet, however, is associated with decreased progression at both stages (Agrón et al. 2022, Keenan et al. 2020, Merle et al. 2019).

### 2.8. Prediction of Future Geographic Atrophy Progression Using the Combination of Prognostic Factors

Several studies have sought to understand the combined contribution of clinical, demographic, and genetic factors to the progression of GA. A combined analysis of the FAM study and the AREDS revealed significant and independent associations of three factors with GA lesion progression. These factors included two genetic factors (*ARMS2_rs10490924* and *C3_rs2230199*) and one clinical component (the presence of GA in the fellow eye). Together, these correlations accounted for up to 7.2% of the observed interindividual variance in GA lesion progression (Grassmann et al. 2015).

It is worth noting that an individual’s prior progression rate can serve as a relatively robust prognostic indicator for their future progression rate (Sunness et al. 2007). Working from the AREDS data, Lindblad et al. (2009) observed a linear model of lesion progress when modeling progression for baseline lesion sizes equal to or larger than 0.5 DA, defined as ≥1.33 mm^2^.

Furthermore, another analysis incorporating data from the FAM and DSGA studies identified key variables, such as lesion area, circularity, perimeter, and caliper diameters. These variables, along with the FAF phenotype, were utilized in linear mixed-effects models to predict future square-root progression rates (Pfau et al. 2019). This analysis demonstrated that shape-descriptive factors accounted for 24.4% of the variance in GA progression among patients without prior observations and 39.1% among those with previous observations, including the consideration of the prior progression rate. These findings underscore the importance of shape-descriptive factors and prior progression as prognostic variables for predicting GA progression. However, it should be noted that a significant portion of the remaining variation in progression appears to be influenced by other variables, some of which can be observed through OCT.

## 3. MEASURES OF RETINAL FUNCTION IN GEOGRAPHIC ATROPHY

This section details the functional metrics used in assessment of GA: BCVA, reading acuity and reading speed, LLVA, microperimetry (MP), electroretinogram, electrooculogram, and patient-reported outcomes.

### 3.1. Visual Acuity Loss in Patients with Geographic Atrophy

The gold standard for assessing treatment efficacy in ophthalmology is BCVA. In patients with GA, gradual loss of RPE and photoreceptors causes a continuous and irreversible decline in BCVA (Chakravarthy et al. 2018, Sunness et al. 1997). Within 18 months of the initial diagnosis of GA, approximately 70% of patients with bilateral GA would not pass the United Kingdom driving standards for visual acuity (Chakravarthy et al. 2018). BCVA declines at a rate of approximately five ETDRS letters annually in patients with GA (Chakravarthy et al. 2018, Heier et al. 2020). However, the rate of decline of BCVA varies widely across patients, and numerous factors can affect this rate.

BCVA is correlated poorly with the total area of GA lesions (Heier et al. 2020, Lindner et al. 2017, Shen et al. 2021c) because, in some eyes, GA spares the fovea until late in the course of the disease, and this measure is highly sensitive to the relative position of GA with respect to the central fovea (Shen et al. 2020, 2021a). An analysis of the AREDS demonstrated that, despite a weak correlation with total GA area (*r*^2^ = 0.07), BCVA was significantly correlated with GA area in the central 1-mm zone (*r*^2^ = 0.45) (Shen et al. 2021c). Eyes with noncentral GA have faster BCVA decline than eyes with central GA, likely because many eyes with central GA have limited residual central area to lose (Shen et al. 2021c). Thus, the determination of GA location is crucial for predicting visual outcomes in the natural history of GA, and this location must be taken into account in the design of studies related to this disease.

Other aspects of GA configuration can also affect the decline rate of BCVA. An analysis of the Chroma and Spectri clinical trials (ClinicalTrials.gov identifiers NCT02247479 and NCT02247531, respectively) found that the decline in BCVA was more pronounced in eyes with unifocal versus multifocal GA and in eyes with smaller versus larger lesions (Heier et al. 2020). Additionally, the trajectory of BCVA loss appears to slow down over time (Sunness et al. 1999). Sunness et al. (1999) reported a three-line BCVA loss in 31% of patients over the first 2 years of a natural history study and in an additional 22% of patients over the subsequent 2 years (Sunness et al. 1999). The AREDS, with over 10 years of follow-up data, showed a similar trend. Importantly, a floor effect is present in GA in which the BCVA plateaued at approximately 45 letters (Lindblad et al. 2009, Shen et al. 2021c). This observation may be explained by the fact that, as GA expands over time, the eye has less and less central vision to lose.

The rate of BCVA decline is also associated with the baseline BCVA. Not surprisingly, the rate of decline of BCVA is faster in the better-seeing eye compared to the worse-seeing eye within the same individual patient (Chakravarthy et al. 2018, Sunness et al. 2008). A prospective natural history study found that patients whose baseline BCVA was 20/50 or more had a 40% chance of losing at least three lines of BCVA over 2 years, but patients with poorer baseline BCVA had a chance of only 13% (Sunness et al. 2008). This finding corresponds to a cohort analysis of a multicenter electronic medical record database, which showed that the BCVA decline rate in the better-seeing eye (12.4 ETDRS letters) was approximately twice as rapid as that in the worse-seeing eye (6.1 ETDRS letters over 2 years) (Chakravarthy et al. 2018). It is plausible that the relatively smaller decrease in BCVA observed in the eye with poorer baseline BCVA or the worse-seeing eye may also reflect a floor effect of visual acuity decline.

Lastly, a faster BCVA decline is associated with a larger baseline low-luminance deficit (LLD), defined as the difference between BCVA on a normally illuminated ETDRS chart and LLVA (Rosenfeld et al. 2018, Sunness et al. 2008). The LLD is a measure of foveal cone function in reduced illumination, and a larger LLD could indicate early impaired foveal cone function despite preserved BCVA. LLVA and LLD are further discussed below.

### 3.2. Reading Acuity and Reading Speed

GA can compromise visual function, even when there still is good BCVA, because parafoveal scotomas and central retinal dysfunction can antedate visible atrophy (Sunness et al. 1997).

The assessment of reading performance (reading acuity and reading speed) provides additional functional information beyond BCVA, particularly in eyes with foveal sparing. Eyes with GA and good BCVA have profound decreases in visual function, particularly while reading and in dim lighting (Sunness et al. 1997); foveal sparing is associated with better reading performance (Künzel et al. 2021, Lindner et al. 2017).

Almost all eyes with GA and central scotoma fixate immediately adjacent to the atrophy (Sunness & Applegate 2005, Sunness et al. 1996). Fixation with the scotoma to the right and with the scotoma in a superior pattern were the first and second most common fixation patterns, respectively. Eyes fixating with the scotoma to the left tended to have lower reading rates than eyes fixating with right or superior patterns. The technique adopted (looking to the right or looking up, for example) is likely to be continued over time, with only minor modifications of the degree of eccentric movement needed (Sunness & Applegate 2005).

Reading acuity parallels BCVA at the preferred retinal locus (PRL) for fixation, whereas reading speed provides additional information regarding retinal function in proximity to the PRL (Künzel et al. 2021). A longitudinal analysis suggested that measurements of reading speed are superior to measurements of reading acuity in terms of the ability to detect change over time and provide additional information regarding retinal function in proximity to the PRL (Künzel et al. 2021).

Binocular reading performance is markedly influenced by the better-seeing eye, with little or no contribution from the worse eye (Künzel et al. 2021). Thus, in patients with GA, clinical and low-vision care should be focused primarily toward the better-seeing eye (Künzel et al. 2021). However, in clinical trials, the worse-seeing eye is often selected, making measurement of monocular reading performance mandatory.

For patients with GA, reading speed declines faster in better-seeing eyes compared to worse-seeing eyes (Heier et al. 2020, Sunness et al. 1997, Varma et al. 2018). Interestingly, the maximum reading speed (MRS) is highly correlated with the size of the atrophic area (Sunness et al. 1996, Varma et al. 2018). For eyes with lesions ≥10 mm^2^ (four DA), the proportion reading below a nonfluent level (MRS <40 words/min) rose from 26.5% at baseline to 64.7% by 18 months when compared to eyes with lesions <10 mm^2^ (baseline: 9.3%; 18 months: 7.0%). These findings suggest that baseline GA lesion size and the magnitude of lesion growth are associated with a decline in reading speed over time and support the use of reading speed as an assessment of functional vision in patients with GA (Varma et al. 2018).

In the Chroma and Spectri trials, reading speed declined at a rate of 18 words/min in 1 year, corresponding to a fall of approximately 22% from baseline, while in the same period, BCVA decreased by approximately five letters, and LLVA decreased by approximately three letters (Heier et al.2020).However, reading speed was influenced by floor effects when lesions were large (Heier et al. 2020), similar to the effects reported for BCVA (Sunness et al. 1999, 2020). Because larger GA lesions have been shown to progress more rapidly in terms of growth than smaller lesions, the findings may also be the result of the transition from foveal sparing to foveal involvement (Fleckenstein et al. 2018, Heier et al. 2020).

Reading performance is strongly correlated not only with conventional structural imaging biomarkers, but also with MP sensitivity. In a cross-sectional evaluation, BCVA, GA area in the central ETDRS subfield, and the presence of foveal sparing were found to be associated with both reading speed and reading acuity. In contrast, LLVA and GA area in the inner-right and inner-upper ETDRS subfields were found to be associated with reading speed but not with reading acuity (Künzel et al. 2021), reflecting the ability of measurements of reading speed to provide additional information regarding retinal function in proximity to the PRL.

In conclusion, monocular reading speed in GA eyes seems to reflect visual function better than BCVA and reading acuity. Measuring monocular reading speed allows one to detect change over time and provides additional information regarding retinal function around the fixation point, supporting its application as an outcome measure in clinical studies. Additionally, as the worse-seeing eye is usually randomized, monocular reading speed should be tested separately (Künzel et al. 2021).

### 3.3. Low-Luminance Visual Acuity

LLVA is another measure used in patients with nonexudative AMD. LLVA is assessed by placing a neutral density filter that decreases the amount of transmitted light over the eye under examination and then testing the participant’s ability to identify the letters displayed on a standardized ETDRS visual acuity chart. LLVA is necessarily lower than BCVA, and the difference between the two visual acuity measures represents the LLD. These endpoints are of particular interest because patients with AMD, especially those with GA, often experience worse functional difficulties in dimly lit environments than in bright conditions (Sunness et al. 1997, 2008). The baseline LLD is positively associated with faster BCVA decline and GA progression (Holekamp et al. 2020, Rosenfeld et al. 2018, Sunness et al. 2008). Thus, LLVA and LLD are important endpoints to include in GA trials.

LLVA loss is generally slower than BCVA decline in patients with GA (Heier et al. 2020, Holekamp et al. 2020, Jaffe et al. 2021). For example, in the lampalizumab Chroma and Spectri trials (ClinicalTrials.gov identifiers NCT02247479 and NCT02247531, respectively), Heier et al. (2020) reported that LLVA loss in patients with GA was slower compared to BCVA decline in the sham group (2.7 versus 4.9 ETDRS letters over 48 weeks).The Proxima A trial (ClinicalTrials.gov identifier NCT02479386; 7.6 versus 13.9 ETDRS letters over 24 months) (Holekamp et al.2020) and the GATHER1 study (ClinicalTrials.gov identifier NCT02686658; 3.5 versus 9.3 ETDRS letters over 12 months) (Jaffe et al. 2021) reported similar results in the sham group. However, the FILLY trial (ClinicalTrials.gov identifier NCT02503332) reported an opposite trend, in which the LLVA loss (3.6 ETDRS letters) was higher than the BCVA loss (0.6 ETDRS letters) in the sham group over 12 months (Liao et al. 2020).

Data on factors associated with the decline rate of LLVA are currently sparse in the literature. Heier et al. (2020) reported that LLVA decline was significantly more rapid in eyes with extrafoveal GA (and those with good baseline BCVA) than in eyes with subfoveal GA. One explanation for this observation is that LLVA is limited by floor effects in eyes with extensive GA and low BCVA. An alternative explanation is that involvement in the fovea may have already shifted the PRL to an eccentric locus, and once this occurs, further changes would be less sensitive to GA expansion. Moreover, the decline in LLVA was more pronounced in eyes with subretinal drusenoid deposits than in those without such deposits (Heier et al. 2020). These subretinal deposits extend through the outer nuclear layer and disrupt photoreceptors (Greferath et al. 2016), thus exacerbating the low-light vision loss. Further long-term studies are needed to understand the natural history of LLVA in AMD and GA in particular and to delineate the role of LLVA in determining the efficacy of treatment in clinical trials.

### 3.4. Microperimetry

The diagnostic landscape of ophthalmology has experienced significant evolution in recent years, primarily driven by advancements in retinal imaging technologies. One such technology is MP, a psychophysical visual function test designed to spatially map retinal sensitivity (Fleckenstein et al. 2018). This test is primarily employed to measure the level of response of the retina to varying intensities of light stimuli, ultimately offering crucial insights into retinal function (Fleckenstein et al. 2018). Developed several decades ago, MP has evolved from a complex, difficult-to-use technology to a more user-friendly and precise tool. Today, it is integral to the functional assessment of the retina, primarily due to advancements like the Nidek MP-1, MP-3, and Macular Integrity Assessment (MAIA) 2 systems, which offer features such as eye tracking and registration (Sunness et al. 2007). The eye-tracking feature, in particular, compensates for ocular movements, enabling accurate and consistent interrogation of specific retinal loci, which is critical for longitudinal analyses of changes in sensitivity.

MP distinguishes itself from traditional perimetry by its ability to lock onto specific fundus features and generate longitudinal functional maps of the retina that correlate to specific retinal loci. Because of this locus-by-locus retinal functional evaluation, MP has the ability to interrogate function even after the fovea has been impacted by GA and is therefore not disproportionately affected by foveal involvement. It has been shown to be one of the few or only functional tests that is moderately correlated with GA lesion area cross-sectionally at baseline and week 48 (Holz et al. 2007). Thus, MP has established itself as an important tool in identifying the correlation between functional data and retinal morphology—an essential element when studying progression from intermediate AMD to GA (Sunness et al. 2007).

**Figure 2:**
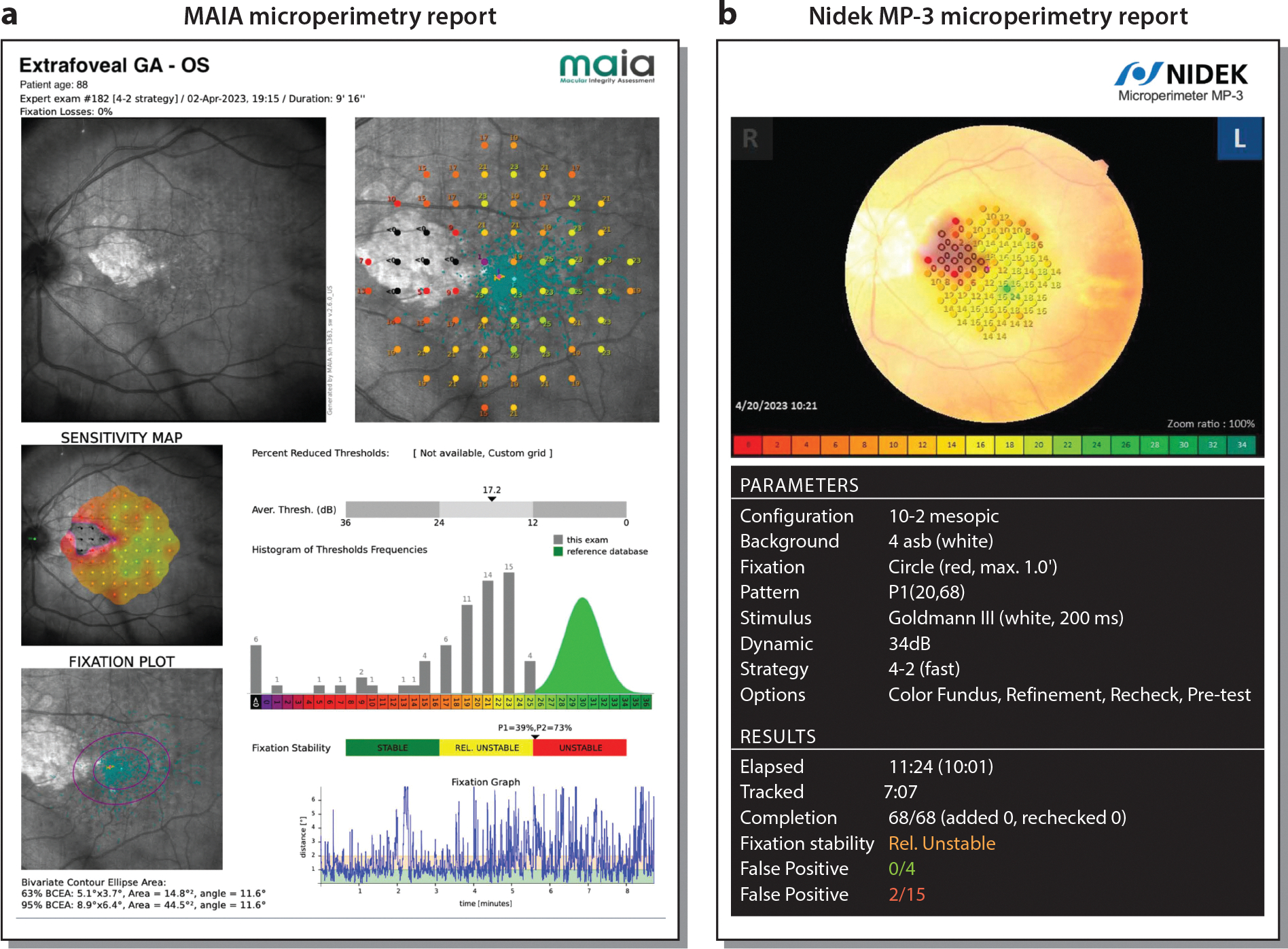
(*a*) Macular Integrity Assessment (MAIA) microperimetry report of an eye with nonsubfoveal geographic atrophy (GA). The parameters were an examination mode of 10–2° Expert Test, a projection strategy of 4–2, and a custom grid of 68 stimuli points covering the central 20°. (*b*) Nidek MP-3 micrometry report of the same eye acquired during the same visit with the same parameters used as the MAIA report. Both sensitivity maps reveal a severe decrease in retinal sensitivity in an area measuring approximately 2.5 × 3.5 mm, corresponding to the area of extrafoveal GA.

There are several strategies for acquiring MP of the retina. Most commonly, a staircase strategy is used, in which light stimuli of decreasing intensities are presented at predetermined loci on the retina. Most MP devices have predetermined grids that interrogate specific loci on the retina. However, most devices also have the flexibility to create custom grids in which different loci can be interrogated based on the need for information. Loci can even be customized on a patient-by-patient basis if so desired, although this strategy may be more logistically cumbersome than the use of standard grids. The test may be completed within 10–15 min but may take longer in an eye with poor fixation issues, where repeated adjustments have to be made by the tracking system or by the operator (Feuer et al. 2013). The test is also operator dependent, since it relies on adequate positioning and instruction of patients, as well as reminding patients to stay focused during the course of the MP. The eventual readout from the device gives the overall mean sensitivity of the tested retinal area (Figure 2).

In eyes with GA, macular sensitivity progressively declines over time as the number of scotomatous points increases (Domalpally et al. 2013), and the average sensitivity of perilesional points decrease at a rate of 1.20 dB/year, a change associated with the expansion of the GA lesion (Domalpally et al. 2013). The decline in sensitivity extends beyond the GA lesion to areas surrounding the lesion (Domalpally et al. 2013, Schmitz-Valckenberg et al. 2016, Shen et al. 2021a). In a study by Schmitz-Valckenberg et al. (2016), decreases in sensitivity were detected in the ring 500 μm adjacent to the lesion, but a steep and acute drop was found at the immediate edge. This precipitous drop in sensitivity seemed to be primarily caused by photoreceptor damage surrounding areas of RPE loss (Shen et al. 2021b). This observation is consistent with postmortem histopathological studies reporting selective loss of rod photoreceptors surrounding GA lesions (Schmitz-Valckenberg et al. 2006, Shen et al. 2020).

One of the challenges in integrating mean MP in GA studies is the fact that if the GA lesion is large, then a large proportion of the retina is nonfunctional on MP and therefore unlikely to change. Therefore, MP points need to lie in the perilesional area, where change is most likely. Several strategies for limiting the locations of MP points have been tested, including patient-customized grids or computational patient-customized analysis of standardized grids. In the latter procedure, the standard 10–2 or other grids are cross-registered with the patients’ baseline fundus images, and a customized perilesional analysis looking at a radius of 250 microns around the lesion border is generated. This approach was used in the Phase 3 OAKS study of pegcetacoplan, an investigational C3 therapy for the treatment of GA, with over 540 patients. A post hoc perilesional analysis of patients tested on a standard 10–2 fovea-centered MAIA mesopic MP grid showed less reduction in retinal sensitivity, and thus loss of function, for patients in the monthly (0.564 dB; *p* = 0.0650) and every-other-month (0.707 dB; *p* = 0.0202) treatment groups compared to the sham group over 24 months (Allingham et al. 2016). Additionally, patients in the every-other month treatment group (−1.138 points; *p* = 0.0140) experienced fewer new scotomatous points compared to those in the sham group over a 24-month period, with the effect increasing over time (Allingham et al. 2016).

The use of MP does come with a set of limitations. One such limitation is that the repeatability of the MP test at the border of a deep scotoma was worse than that at other areas of the normal retina (Biarnés et al. 2015). An additional disadvantage is that the test takes a long time in patients with poor vision. This can be mitigated, at least in the case of GA, by patient-customized grids. However, generating and implementing patient-customized grids can be a challenge, especially in the setting of large-scale clinical trials, where this would both add logistical complexity and possibly increase the chances of operational errors.

### 3.5. Other Visual Functional Measures

Patients with AMD, especially in late stages of the disease, such as GA, display a reduction in retinal function in electrophysiological testing. In a systematic review of six full-field electroretinogram (ffERG) studies in 481 eyes, Forshaw and colleagues (2022) showed that patients with any AMD had prolongation of light-adapted a-wave implicit times representing cone function and reduction in light-adapted b-wave parameters representing ON- and OFF-bipolar cell function. Dark-adapted a- and b-wave amplitudes representing mixed photoreceptor and postreceptor ON-pathway function were also significantly impaired. GA eyes displayed longer rod-driven b-wave implicit times on ffERG compared to other AMD subgroups (Walter et al. 1999). The explanation for this phenomenon may be that the rod dysfunction in AMD is not notable on ffERG until the disease is extensive and has progressed beyond the macula (Forshaw et al. 2021, Quinn et al.2019). Electrooculogram data of the dark trough, light peak, and Arden ratio also showed considerable reductions in all of these measures in AMD patients compared to controls in two separate studies (Kader 2017, Walter et al. 1999).

Patient-reported visual impairments have been reported in AMD. On the Low-Luminance Questionnaire (LLQ), 113 participants affected by a range of AMD disease severity had associations between patient-reported visual deficits with both prolonged rod-intercept time on dark adaptation and reduced choroidal thickness. The rod-intercept association was stronger than choroidal thickness in multivariable analyses. The strongest correlation was between the LLQ driving subscales and dark adaptation. All subscale associations were statistically significant except for that between choroidal thickness and the peripheral vision subscale (Yazdanie et al. 2017).

The NEI-VFQ demonstrated vision-related quality of life (VRQoL) to be significantly impaired in individuals with GA. Künzel and collaborators (2020) showed that the VRQoL composite score was impaired at baseline in 87 GA patients. In a multivariate cross-sectional analysis, BCVA, GA size, and LLVA for the better eye and BCVA, foveal sparing, and LLVA for the worse eye predicted the VRQoL composite score. The best longitudinal predictors of composite score identified in a subset of 66 patients were BCVA, LLVA for the better eye, and GA size. The NEI-VFQ results appear to be impacted by the topographic location of GA. The area of GA in the central 1-mm-diameter zone of the better eye was significantly correlated with VRQoL by linear regression modeling when the ETDRS subfields were examined in 237 eyes from 161 subjects in the AREDS. However, in a multivariable analysis, VRQoL was not associated with total area of atrophy in the better or worse eye, suggesting that topographic location in the better eye is most important for NEI-VFQ measures (Ahluwalia et al. 2023).

The natural progression of VRQoL differs in central GA versus neovascular AMD (nAMD), as shown in a study of 404 participants from AREDS that calculated the rates of change of VRQoL as the slopes of linear models fit to longitudinal individual-level NEI-VFQ scores. Prior to the development of advanced AMD, the rate of VRQoL decrease was greater for participants that eventually developed central GA versus those that developed nAMD. After progression to advanced AMD, the slope was greater in nAMD compared with central GA. Notably, female gender and a higher baseline VRQoL score were independently associated with a larger longitudinal decline in VRQoL following progression to advanced AMD (Ahluwalia et al. 2023).

## 4. DESIGN OF FUTURE GEOGRAPHIC ATROPHY TRIALS

Endpoint discordance in GA trials is a major issue when it comes to estimating the impact of a beneficial effect that has been unequivocally demonstrated in an anatomical outcome but that does not have a corresponding advantage in a functional measure. This was exemplified in the recent large anticomplement trials conducted in the GA space, which have reported between 15% and 20% reductions in GA growth in treated eyes within the first year of treatment initiation, increasing to as much as 30% reduction in the second year (Heier et al. 2023, Khanani et al. 2023, Liao et al.2020).The robustness of the mitigation by treatment of GA expansion is convincing due to the consistency of the effect from Phase 2 to Phase 3 within one of the agents tested across trials, with disparate molecules acting within the same biological pathway, and due to the enhancement of the benefit seen going from year 1 into year 2 with continuing treatment.

None of these trials have shown a difference in the slope of functional loss in prespecified outcome measures between the treated and sham groups over time. The treatment itself is invasive, with repeated intravitreal administration of the therapeutic. The rare side effects that have been reported with these treatments carry a serious risk of vision loss due to potential endophthalmitis and optic neuritis. However, the more common cause of concern is the potential for onset of exudation in the macular region of the treated eye, which, although manageable with the anti-VEGF agents, is a possible cause of worsening vision loss over time (Heier et al. 2023, Liao et al. 2020). Furthermore, such a scenario will result in additional invasive treatment procedures and extra visits and investigations, creating conditions for more adverse events while also increasing costs and the burden of care to the patient and the provider. The discussion above emphasizes the need to demonstrate a high benefit-to-risk ratio in terms of function, as this is the critical element for the patient and for payors in both cost-effectiveness and cost-utility calculations.

In another large trial of GA, in which the drug lampalizumab failed to show efficacy compared to sham, a secondary treatment-agnostic analysis with the aim of reporting associations between function and the morphology of the GA lesion was performed. The authors reported a clear lack of correlation between the change in area of GA and the change in a plethora of measures of visual function that included BCVA, reading speed, LLVA, and MP (Heier et al. 2020). Correlations were either weak or modest, with retinal sensitivity measured by MP demonstrating the best performance. Given that none of the measures of function showed a sufficiently strong correlation with the change in GA area, the absence of any differential in treatment groups in the latest GA trials is unsurprising. To understand this lack of treatment response in function despite the clear benefit in the anatomical endpoint, it is worth considering some of the findings from the lampalizumab analyses. Differences in the trajectory of functional decline of BCVA were observed among the treatment groups, some of which showed characteristics of GA, including foveal involvement, focality, size, area of lesion, and presence of pseudodrusen. Interestingly, the decline in BCVA was similar in subfoveal and extrafoveal lesions, although the latter group exhibited a marginally steeper fall in LLVA and reading speed over 2 years. Unifocal lesions consistently experienced greater falls in most of the tested measures of visual function, and smaller lesions also fared worse compared to larger lesions. As discussed in the preceding sections, extrafoveally located larger lesions are faster growing and would constitute the best group to include in trials when anatomical endpoints are used. This is of particular interest since the eligibility criteria for inclusion, the sample size and the anticipated difference between treatment and sham groups, are based on enrolling eyes with higher GA expansion rates. However, the trajectory of visual decline in such eyes can be shallow, particularly with BCVA, which remains the most commonly used, most practical, most easily performed, and best understood measure of function. Nonetheless, the information that has been gleaned from prior data analyses suggests that, within large data sets, there will exist subgroups of patients whose study eyes will fit the criteria for rapid growth of the lesion and also exhibit faster reductions in BCVA. In such a sample of study eyes, there is the potential to show correspondence between anatomical and functional benefit.

The AREDS reported that, among eyes with GA without foveal involvement at first presentation, the lesions became subfoveal within 2.4 years on average. Patients may compensate by using residual functioning retina through eccentric fixation. However, the PRL (Bernard & Chung 2018, Sunness et al. 2000) can move and also vary with the task, leading to variability when testing function over time. Nonetheless, the use of categorical outcomes based on functional tests accompanied by time-to-event analyses may help capture the proportions with persistent vision loss within high-risk subgroups. Thus, for example, in the GATHER trials, the investigators used an endpoint of 15 letters lost that persisted over two visits as a categorical outcome event. Applying this technique, Danzig et al. (2023) found a 56% reduction in the relative risk of persistent vision loss in the ACP 2-mg treated group compared to sham. Similarly, in the OAKS and DERBY trials, which included tests of macular sensitivity, the increase in the number of scotomatous endpoints was lower in the treatment arms compared to the sham arm.

## 5. SUMMARY

Although unraveling the relationships between GA progression and visual loss has been challenging, the past decade has yielded many studies creating opportunities to identify the characteristics of subgroups of GA-affected eyes that have a high risk of both anatomical expansion and concomitant visual acuity loss. Using this information and revised analytical techniques, it has been possible to reveal functional benefits when GA expansion has been slowed. To date, these analyses have been post hoc and restricted to small samples. Future GA trials can use this information to define eligibility criteria, calculate the sample size, and provide an analytical plan with the direction and size of the effect prespecified. Such a strategy would go a long way toward convincing clinicians and payors of the value of treatment.

## SUMMARY POINTS

1. Recent clinical trials of anticomplement therapies have shown modest yet consistent reductions in geographic atrophy (GA) growth but no commensurate changes in visual function.
2. Several studies have described the characteristics of GA morphology, the factors influencing growth rates of the lesion and directionality of expansion, and the various measures of vision that reflect changes in function in GA.
3. The design of future trials should be made more targeted by taking into consideration both the factors that modify GA expansion and those that influence functional decline to align these key outcomes and convince clinicians and payors of the value of treatment.

## FUTURE ISSUES

1. To demonstrate differences in GA growth rate, future clinical trials should continue to enrich for larger, rapidly expanding multifocal and extrafoveal GA lesions, as was done in the recent Phase 3 registration trials inhibiting the alternative complement pathway.
2. For the currently used measures of visual function to adequately capture the disability experienced by GA patients in clinical trials, these studies should enrich for populations that have not been targeted previously. Specifically, these studies should enroll participants with good to moderate visual function and smaller unifocal subfoveal GA lesions.
3. The design of a future trial of GA with the ability to show a meaningful difference in both structural and functional outcomes is challenging but is expected to be feasible if the correct population is considered.
4. As a next step, researchers should consider earlier stages of dry age-related macular regeneration for therapeutic intervention. Through a variety of mechanisms of action, this approach would allow the prevention of functional decline before the onset of irreversible retinal changes.
